# TWEAK increases CD74 expression and sensitizes to DDT proinflammatory actions in tubular cells

**DOI:** 10.1371/journal.pone.0199391

**Published:** 2018-06-20

**Authors:** Lara Valiño-Rivas, Leticia Cuarental, Osvaldo Grana, Richard Bucala, Lin Leng, Ana Sanz, Gonzalo Gomez, Alberto Ortiz, Maria Dolores Sanchez-Niño

**Affiliations:** 1 IIS-Fundacion Jimenez Diaz, Madrid, Spain; 2 Bioinformatics Unit, Structural Biology and Biocomputing Programme, Spanish National Cancer Research Centre, Madrid, Spain; 3 Department of Medicine, Yale University, New Haven, Connecticut, United States of America; Hopital Tenon, FRANCE

## Abstract

CD74 is a multifunctional protein and a receptor for Macrophage Migration Inhibitory Factor (MIF) and MIF-2 / D-dopachrome tautomerase (DDT) cytokines, upregulated in diabetic kidney disease. However, the drivers of CD74 expression and DDT function in kidney cells are poorly characterized. TWEAK is a proinflammatory cytokine that promotes kidney injury. We have now identified CD74 gene expression as upregulated in the kidneys in response to systemic TWEAK administration in mice, and have characterized the in vivo CD74 expression and the functional consequences in cultured cells. TWEAK administration to mice resulted in a progressive time-dependent (up to 24h) upregulation of kidney CD74 mRNA (RT-PCR) and protein (Western blot). Furthermore, the CD74 ligands MIF and DDT were also upregulated at the protein level 24h after TWEAK administration. Immunohistochemistry localized the increased CD74, MIF and DDT expression to tubular cells. In cultured tubular cells, TWEAK increased CD74 mRNA and protein expression dose-dependently, with a temporal pattern similar to in vivo. TWEAK-induced CD74 localized to the cell membrane, where it can function as a cytokine receptor. For the first time, we explored the actions of DDT in tubular cells and found that DDT amplified the increase in MCP-1 and RANTES expression in response to TWEAK. By contrast, DDT did not significantly modify TWEAK-induced Klotho downregulation. In conclusion, TWEAK upregulates CD74 and its ligands MIF and DDT in renal tubular cells. This may have functional consequences for kidney injury since DDT amplified the inflammatory response to TWEAK.

## Introduction

Tumor necrosis factor-like weak inducer of apoptosis (TWEAK) is a proinflammatory cytokine of the TNF superfamily that activates the fibroblast growth factor-inducible-14 (Fn14) receptor [1–3] as reviewed in detail previously [1]. TWEAK actions on intrinsic kidney cells and on inflammatory cells may contribute to kidney injury. Thus, in cultured tubular cells TWEAK induces the expression of inflammatory cytokines, downregulates the expression of the anti-aging and anti-inflammatory factor Klotho, is mitogenic, and in the presence of sensitizing agents, promotes apoptosis [1–5]. Increased expression of TWEAK and Fn14 was reported in human and experimental acute and chronic kidney disease [6,7]. Indeed, the role of TWEAK/Fn14 in kidney injury has been demonstrated in functional studies using anti-TWEAK antibodies or genetically modified mice in diverse forms of experimental acute kidney injury and chronic kidney disease (CKD) [8–13]. However, the molecular mechanisms involved in the deleterious effect of TWEAK in kidney disease are still incompletely understood.

CD74 (MHC class II invariant chain, Ii) is a transmembrane glycoprotein that regulates intracellular protein trafficking as a chaperone and is the cognate cell surface receptor for the cytokines macrophage migration inhibitory factor (MIF) and D-dopachrome tautomerase (D-DT/MIF-2) [14,15], as reviewed in detail previously [16]. During kidney injury, leukocytes and intrinsic renal cells such as podocytes and tubular epithelial cells express CD74 [17]. In the kidneys, MIF promotes experimental glomerular injury and cystogenesis [17,18]. Furthermore, CD74 deficient mice are protected from glomerular injury induced by anti-GBM antiserum [19]. CD74 modulates B cell, T cell and dendritic cell responses [14,15] and milatuzumab, an anti-CD74 antibody, has orphan drug status for the treatment of multiple myeloma and chronic lymphocytic leukemia [20]. In renal cells, MIF activates CD74 to promote a proinflammatory response [21]. In this regard, CD74 may modulate tissue injury and homeostasis beyond its effect on immune regulation.

CD74 expression is increased during tissue injury in diverse organs and in malignancies, including kidney cancer [16,17,22]. In normal mouse and human kidneys, tubular but not glomerular epithelium express low levels of CD74 [21]. CD74 is upregulated in tubular epithelial cells and/or podocytes during diverse human kidney diseases [18,21,23,24].

While MIF has been implicated in glomerular and tubulointerstitial injury [16,17], very little is known about D-dopachrome tautomerase (DDT), a second ligand for CD74, in kidney disease [16,25]. In addition, the factors regulating CD74 or DDT expression in kidney cells are poorly characterized. Understanding these factors may help modulate the impact of CD74 or DDT in kidney injury. We have now explored the regulation of CD74 and DDT expression by TWEAK in kidney cells and the functional consequences of this regulation.

## Material and methods

### Animal model

All animal work have been conducted according to national and international guidelines and was approved by the Fundacion Instituto Investigacion Sanitaria Fundacion Jimenez Diaz animal research ethics committee. Euthanasia was performed by cervical dislocation.

Studies were conducted in accord with the NIH Guide for the Care and Use of Laboratory Animals. Female, 12- to 14-week-old C57/BL6 mice from the IIS-Fundacion Jimenez Diaz animal facilities were administered 0.75 μg TWEAK or saline intraperitoneally and were killed 4 and 24 h after injection (n = 5 per group). The dose of TWEAK was calculated on the basis of cell culture dose-response experiments for an extracellular volume of 7.5 ml/mouse and was previously shown to elicit biological responses in vivo [26]. Kidneys were perfused in vivo with ice-cold saline and processed for immunohistochemistry or immediately frozen for RNA and protein studies.

### Cells and reagents

MCT cells are a cultured line of proximal tubular epithelial cells harvested originally from the renal cortex of SJL mice and have been extensively characterized [27]. They were cultured in RPMI 1640 (GIBCO, Grand Island, NY, USA), 10% decomplemented fetal bovine serum (FBS), 2 mM glutamine, 100 U/ml penicillin and 100 μg/ml streptomycin, in 5% CO2 at 37 °C [27]. Recombinant human soluble TWEAK (Millipore, Billerica, MA) was used at 10 to 100 ng/ml, based on prior dose-response experiments [5]. DDT (Prof. Bucala, Yale University School of Medicine) was used at 10 ng/mL based on prior experience activating the CD74 receptor on renal cells with MIF and on circulating levels of DDT [21]. The NFκB inhibitor parthenolide (Sigma, St. Louis, MO) was used at 10 μM based on previous dose-responses studies [28].

### Western blot analysis

Tissue and cell samples were homogenized in lysis buffer, separated by 10% or 12% SDS-PAGE under reducing conditions and transferred to PVDF membranes (Millipore, Bedford, MA, USA), blocked with 5% skimmed milk in PBS/0.5% v/v Tween 20 for 1 h, and washed with PBS/Tween [28]. Primary antibodies were rabbit polyclonal anti-CD74 (1:500, Santa Cruz, CA, USA), anti-MIF (1:500, Santa Cruz, CA, USA) and anti-DDT (1:500, Abcam). Antibodies were diluted in 5% milk PBS/Tween. Blots were washed with PBS/Tween and subsequently incubated with appropriate horseradish peroxidase-conjugated secondary antibody (1:2000, GE Healthcare/Amersham, Aylesbury, UK). After washing, blots were developed with the chemiluminescence method (ECL). Blots were then re-probed with monoclonal anti- mouse α-tubulin antibody (1:2000, Sigma) and levels of expression were corrected for minor differences in loading.

### Quantitative reverse transcription-polymerase chain reaction

One μg RNA isolated by Trizol (Invitrogen, Paisley, UK) was reverse transcribed with High Capacity cDNA Archive Kit and real-time PCR was performed on a ABI Prism 7500 PCR system (Applied Biosystems, Foster City, CA) using the DeltaDelta Ct method [28]. Expression levels are expressed as ratios to GAPDH. Pre-developed primer and probe assays were from Applied Biosystems.

### Immunohistochemistry

Immunohistochemistry was carried out as previously described on paraffin-embedded 5 μm thick tissue sections [27]. Primary antibodies were rabbit polyclonal anti-CD74 (1:50, Santa Cruz, CA, USA), anti-DDT (1:100, Abcam) and anti-MIF (1:100, Santa Cruz). Sections were counterstained with Carazzi`s hematoxylin. Negative controls included incubation with a non-specific immunoglobulin of the same isotype as the primary antibody. Sections were subsequently incubated with the proximal tubule marker, fluorescein-conjugated tetragonolobus lotus lectin (1:33, Vector Lab, Peterborough, United Kingdom). Staining was evaluated by a quantitative scoring system, Image-Pro Plus software (Media Cybernetics, Bethesda, MD) in 10 randomly selected fields (x20) per kidney. Samples were examined in a blinded manner.

### Flow cytometry analysis of cell surface CD74 expression

Cells were detached with 2 mM EDTA and 5 x 10^5^ cells were incubated for 30 min at 4°C with 8 μg/ml rabbit anti-CD74 antibody (Santa Cruz) or control IgG followed by a 30-min 4°C incubation with 1:100 FITC secondary antibody (Pharmingen, San Diego, CA) [29]. Mean cell fluorescence was calculated using Cell Quest Software (Becton Dickinson, Franklin Lakes, NJ).

### Statistics

Statistical analysis was performed using SPSS 11.0 statistical software (IBM, NY, USA). Results are expressed as mean ± SEM. Significance at the p <0.05 level was assessed by Student´s t test for two groups of data and ANOVA for three of more groups with Bonferroni correction.

## Results

### TWEAK increases CD74 expression in tubular cells in vivo

**Fig 1:**
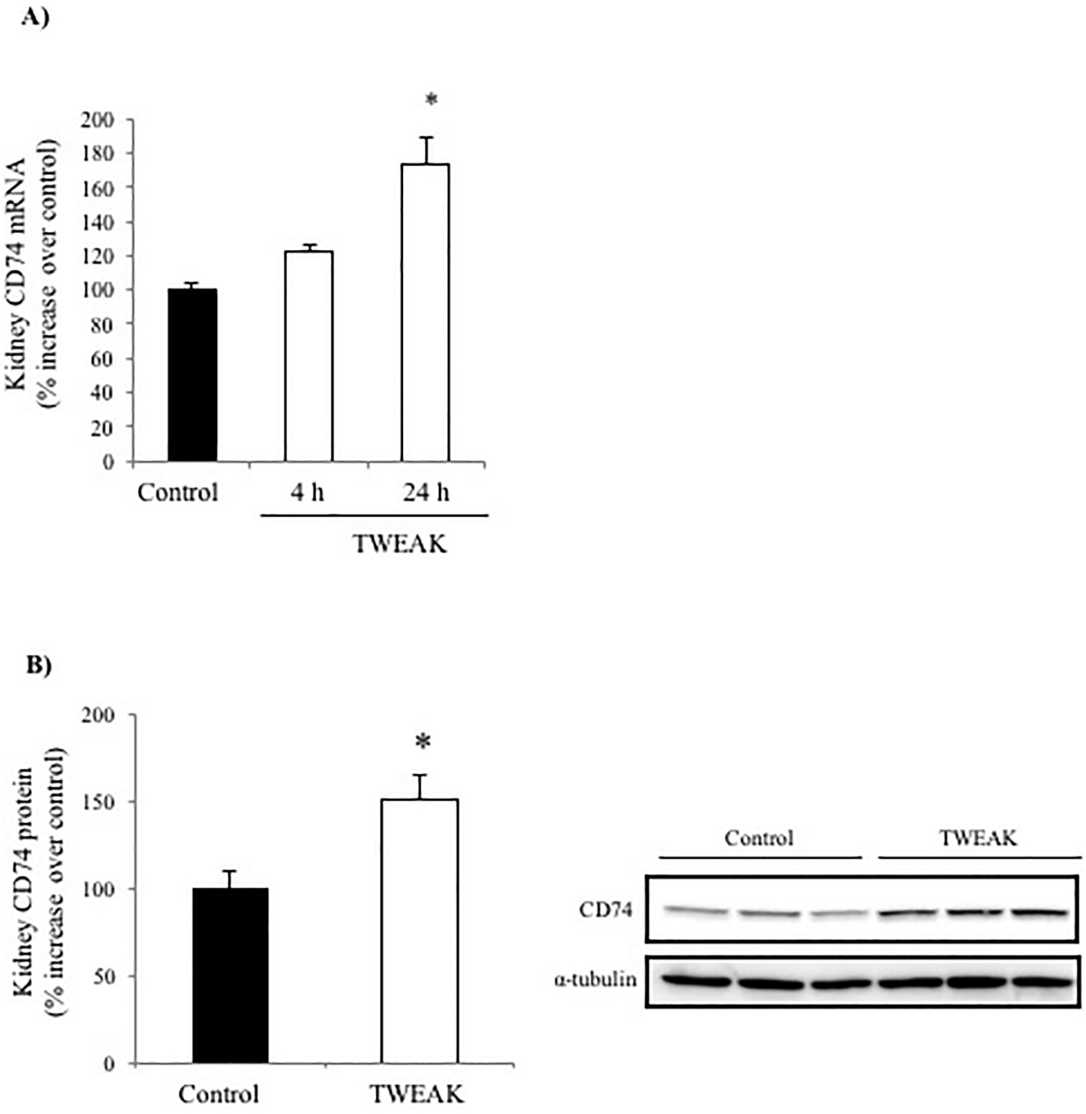
TWEAK increases CD74 expression in tubular cells in vivo. **A)** Quantitative RT-PCR analysis of whole kidney CD74 mRNA in mice 4 h and 24 h after TWEAK or vehicle injection. *p<0.005 versus control. **B)** Whole kidney CD74 protein expression 24 h after TWEAK or vehicle injection. Quantification of Western blot and representative image. *p<0.005 versus control. Data are mean ± SEM of 5 mice per group.

**Fig 2:**
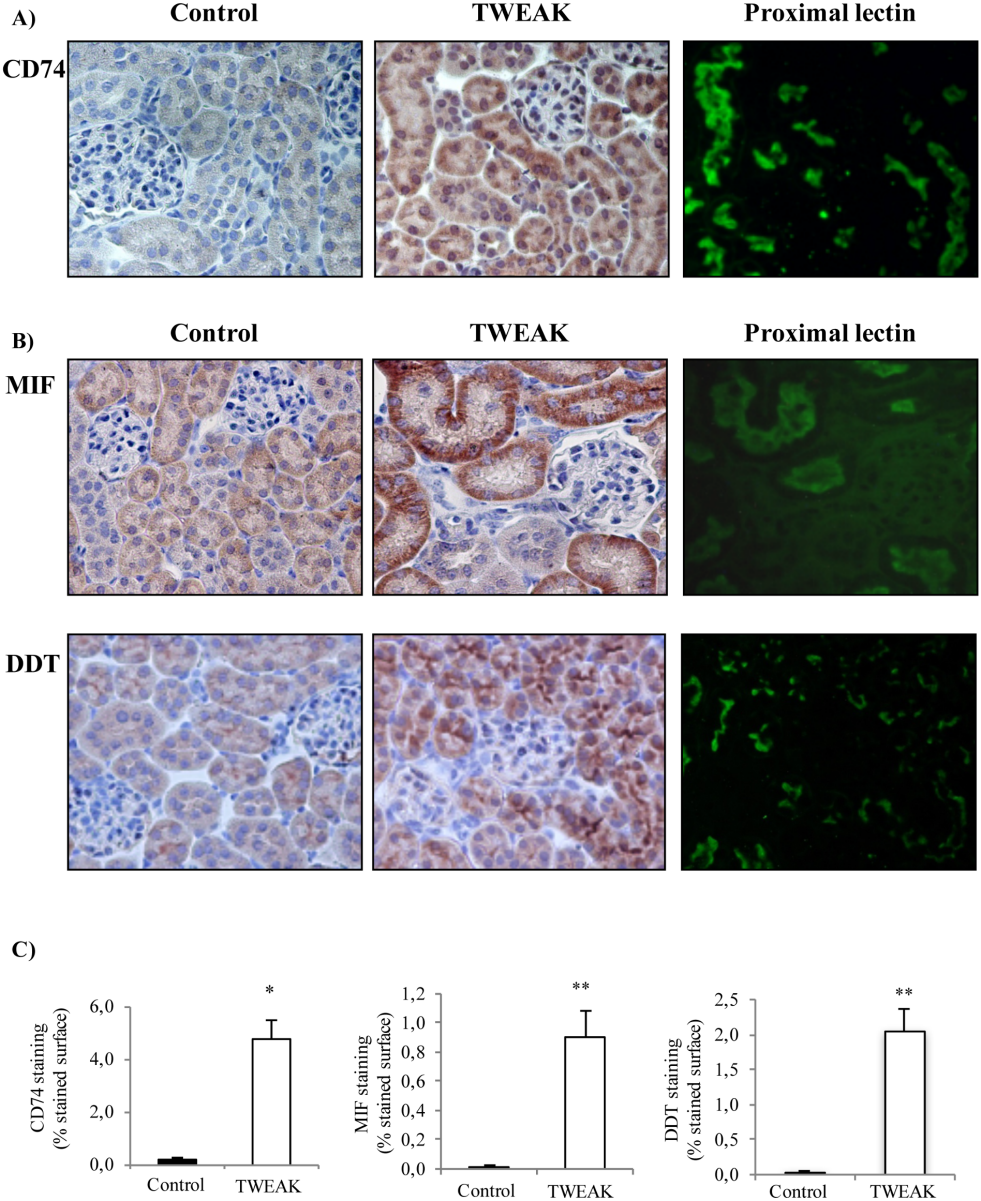
Increased expression of CD74, MIF and DDT following TWEAK administration is localized to tubular cells. **A)** CD74 immunohistochemistry localized the increased CD74 expression to tubular cells 24 hours after TWEAK or vehicle injection (arrow). **B)** MIF and DDT immunohistochemistry localized their increased expression to tubular cells 24 hours after TWEAK or vehicle injection. Tetragonolobus lotus lectin (green) stains proximal tubular cells and colocalized in tubules with the three proteins. Original magnification x400. Images representative of 5 animals per group. **C)** CD74, MIF and DDT quantification expressed as mean mean ± SEM *p<0.03 versus control, **p<0.003 versus control.

**Fig 3:**
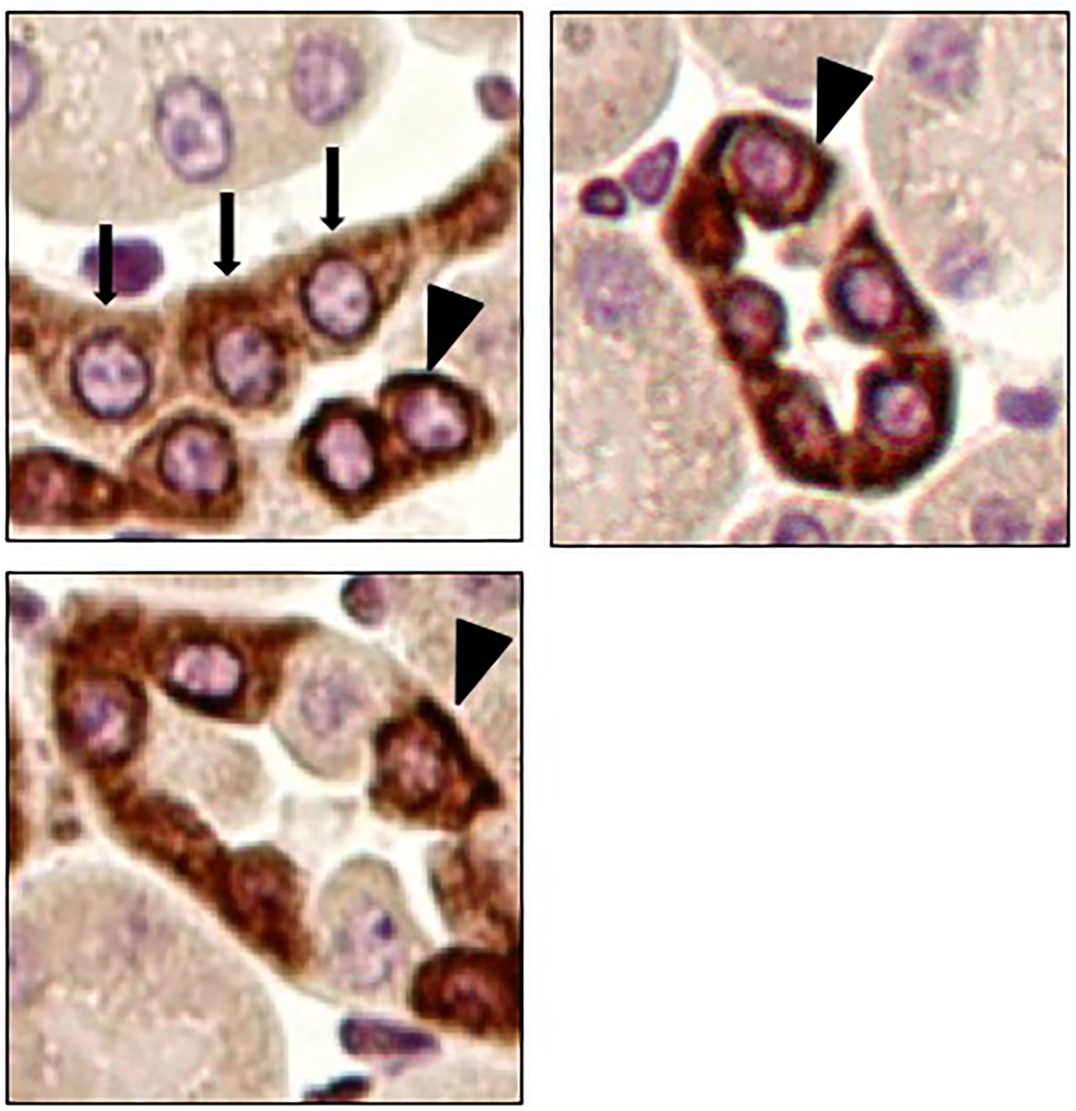
CD74 localization to perinuclear and cell membrane areas in distal tubular cells. CD74 immunohistochemistry localized the increased CD74 expression to tubular cells 24 hours after TWEAK injection to perinuclear (arrows) and cell membrane areas (arrowheads). Original magnification x400.

Systemic TWEAK administration promotes an inflammatory response in the kidneys that is already evident 4h after TWEAK administration [5]. Increased CD74 expression following TWEAK administration was observed by qRT-PCR: TWEAK-induced upregulation of kidney CD74 mRNA was already present at 4 h and peaked at 24 h (Fig 1A). Furthermore, TWEAK-induced upregulation of CD74 protein was observed at 24 h (Fig 1B). Immunohistochemistry localized the increased CD74 expression to tubular epithelium (Fig 2A). Specifically, proximal tubule lectin staining localized CD74 to proximal tubular cells (Fig 2A), although the highest CD74 expression located to distal tubules (Fig 3). The increased CD74 expression localized both to the perinuclear area, a typical CD74 expression pattern, and also to the cell membrane area, where it could serve as a DDT or MIF receptor (Fig 3).

### TWEAK increases MIF and DDT expression in tubular cells in vivo

**Fig 4:**
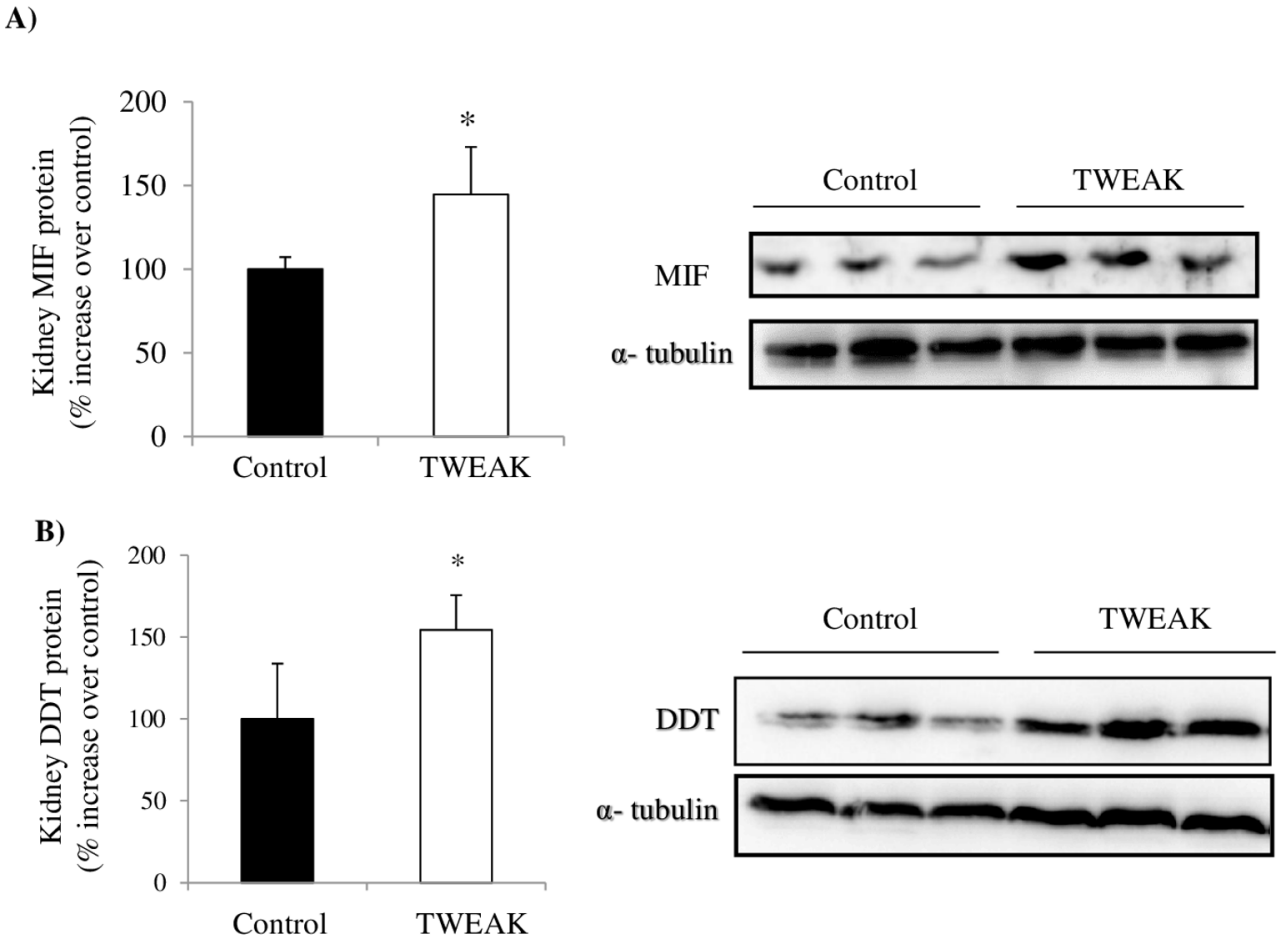
TWEAK increases kidney MIF and DDT protein levels in vivo. **A)** Whole kidney MIF and **B)** DDT protein expression 24 hours after TWEAK or vehicle injection. Quantification of Western blot and representative image. *p<0.01 versus control. Data are mean ± SEM of 5 mice per group.

MIF and DDT are the two known ligands for CD74. TWEAK also upregulates kidney MIF and DDT protein at 24 h in vivo (Fig 4A and 4B). Immunohistochemistry localized the expression of MIF and DDT to tubular cells (Fig 2B and 2C). Specifically, proximal tubule lectin staining localized both proteins to proximal tubular cells (Fig 2B and 2C). DDT and MIF were located on opposite poles of the cell, potentially suggesting secretion to different compartments. Thus, TWEAK upregulated both the two ligands and the receptor within the same cell compartment (tubular cells) and at the same time point. Under the staining conditions, no upregulation of CD74, MIF or DDT was apparent in glomeruli (Fig 2A–2C).

### TWEAK increases CD74 and DDT expression in cultured tubular cells

**Fig 5:**
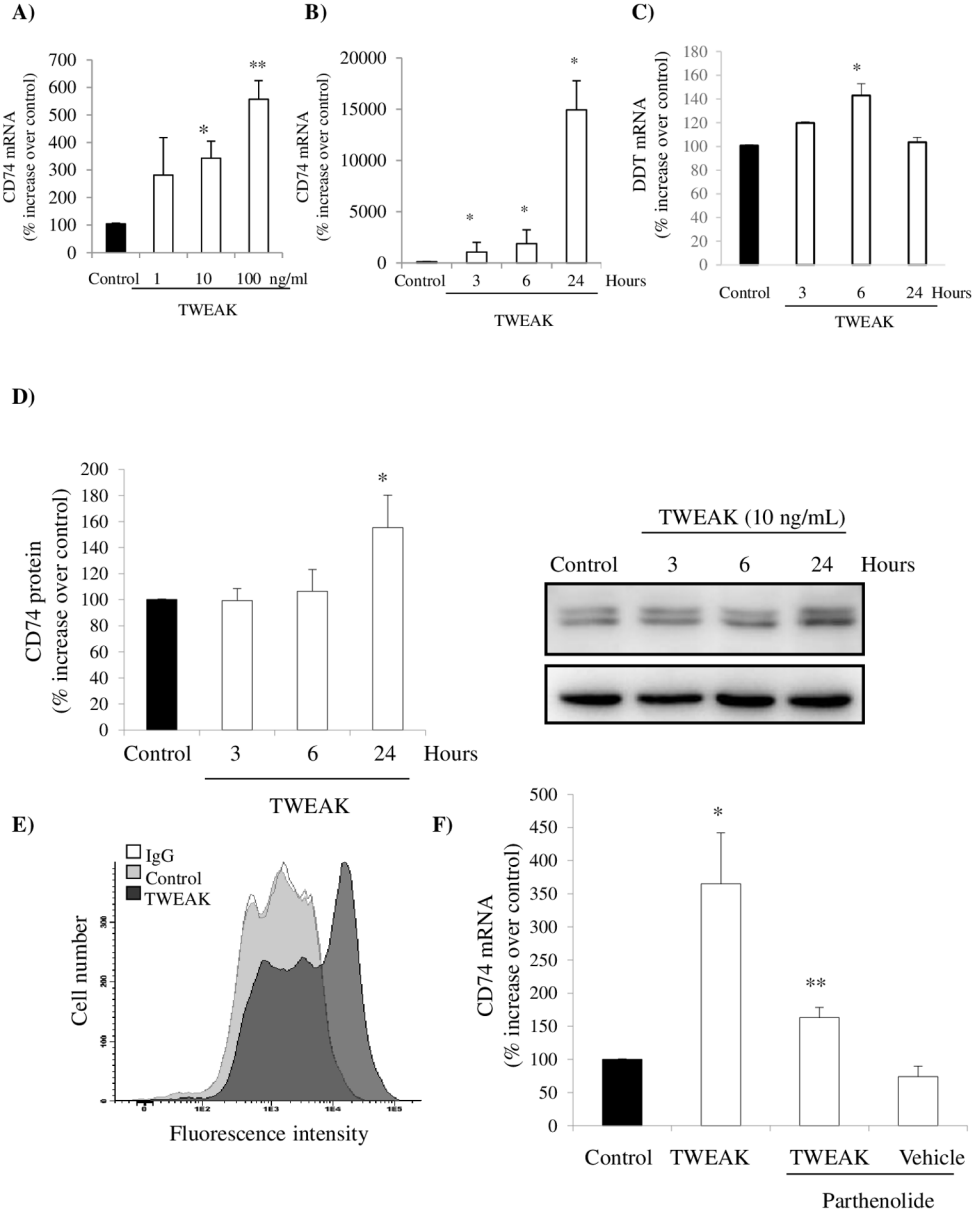
TWEAK increases CD74 and DDT expression in cultured tubular cells. **A)** Dose-response of CD74 mRNA induction at 3h. mRNA expression was assessed by qRT-PCR. *p<0.01 vs control, **p<0.001 vs vehicle control. **B)** Time-course. Cells were stimulated with 10 ng/mL TWEAK and CD74 mRNA expression was assessed by qRT-PCR. *p<0.002 vs control. **C)** Time-course. Cells were stimulated with 10 ng/mL TWEAK and DDT mRNA expression was assessed by qRT-PCR. *p<0.01 vs control. **D)** Time-course. Cells were stimulated with 10 ng/mL TWEAK and CD74 protein in whole cell lysates was assessed by Western blot. *p<0.03 vs control. **E)** Flow cytometry of non-permeabilized tubular cells showed that CD74 expression is increased in the cell surface 24 h after 10 ng/mL TWEAK stimulation as compared to vehicle control. IgG denotes cell stained with non-immune IgG. Control and TWEAK samples were stained with anti-CD74 antibody. **F)** Cells were stimulated with 10 ng/mL TWEAK for 3 hours. TWEAK-induced CD74 mRNA expression was prevented by parthenolide. *p<0.002 vs control; **p<0.007 vs TWEAK. Data are mean ± SEM of four independent experiments.

Once tubular cells were identified in vivo as expressing CD74 and DDT following TWEAK stimulation, we assessed TWEAK regulation of CD74 and DDT expression in cultured tubular cells. In these cells, TWEAK induced upregulation of CD74 and DDT mRNA in a dose-dependent manner (Fig 5A). Similar to the in vivo findings, CD74 mRNA expression increased progressively over 24h (Fig 5B). TWEAK also increased DDT mRNA in a time-dependent manner (Fig 5C). Also consistent with the in vivo findings, TWEAK-induced upregulation of CD74 protein was observed at 24 h (Fig 5D).

The bulk of CD74 is usually confined to the intracellular perinuclear region [30]. In non-permeabilized tubular cells, flow cytometry assessment of cell surface CD74 confirmed that TWEAK-induced upregulation of CD74 was associated with increased cell surface CD74 (Fig 5E). At this location, CD74 can function as a receptor for MIF and DDT.

In tubular cells, TWEAK activates several signal transduction pathways that converge at the transcription factor NF-κB, leading to nuclear translocation of the key component of the canonical NF-κB activation pathway, NF-κB p65 [5,31,32]. In prior studies, we have shown that parthenolide inhibits TWEAK-induced NF-κB DNA-binding activity and subsequent NF-κB p65-dependent gene transcription [5]. In this regard, TWEAK-induced upregulation of CD74 mRNA expression was abrogated by parthenolide (Fig 5F), indicating that it is a canonical NF-κB activation–dependent response.

### TWEAK sensitizes tubular cells to DDT proinflammatory actions

**Fig 6:**
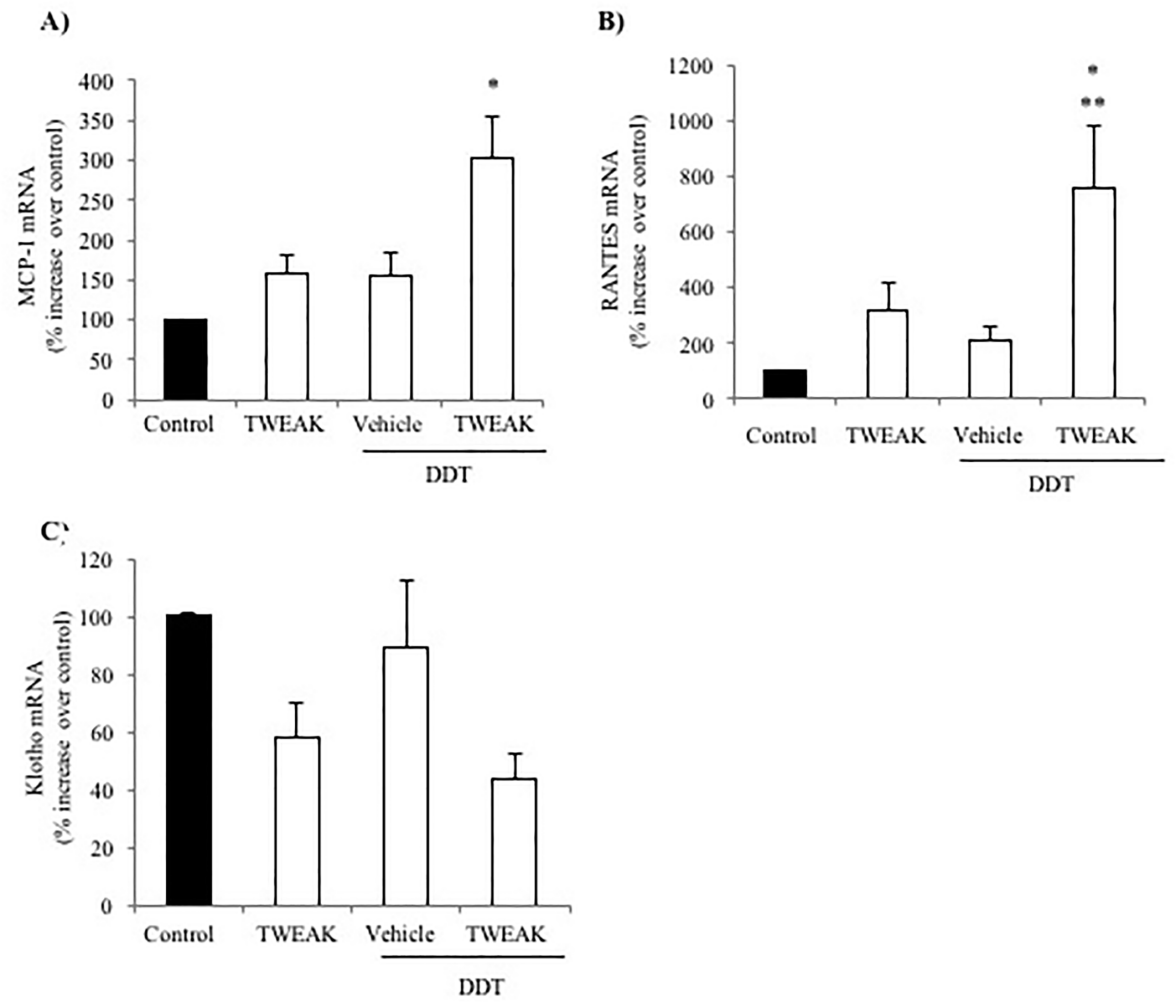
TWEAK sensitizes to DDT proinflammatory actions in tubular cells. Cells were stimulated with 10 ng/mL TWEAK for 3 hours and then with 10 ng/mL DDT for 24 hours. **A)** MCP-1 and **B)** RANTES mRNA was increased after DDT stimulation in cells pretreated with TWEAK. **C)** Under the same experimental conditions, Klotho mRNA was downregulated after TWEAK stimulation but addition of DDT had no statistically significant effect over TWEAK alone. Expression of mRNA was assessed by real time RT-PCR. *p<0.02 vs control, **p<0.05 vs DDT. Mean ± SEM of four independent experiments.

Since TWEAK upregulated the expression of CD74 and DDT in vivo, we explored the functional consequences of this observation. The function of DDT in kidney cells had not been previously explored. Under basal conditions, DDT had a very mild proinflammatory activity in cultured tubular cells that did not reach statistical significance (Fig 6A–6C). This may be related to the low constitutive cell surface CD74 expression (Fig 5D). However, increasing CD74 expression by pre-stimulating with TWEAK resulted in a large increase in the expression of MCP-1 and RANTES mRNA in response to DDT, well above the expression elicited by either DDT or TWEAK alone (Fig 6A and 6B). By contrast, DDT did not influence the expression of Klotho mRNA and did not significantly modify TWEAK-induced Klotho downregulation (Fig 6C).

## Discussion

The main findings are that TWEAK promotes the expression of CD74 and DDT in tubular cells, and increases the availability of CD74 at the cell surface, thus sensitizing to the proinflammatory action of DDT. This information is useful to design therapeutic strategies aimed at modulating CD74 expression both in kidney disease and outside the kidney, and as well as to design strategies to protect the kidneys from the therapeutic use of anti-CD74 antibodies, as discussed below.

Functional preclinical in vivo studies have shown that TWEAK is a key mediator of several forms of kidney injury [1]. However, the molecular mediators recruited by TWEAK remain poorly understood. CD74 has several functions, including being a receptor for MIF and DDT [16,17]. We have centered the study on persistent (24h) changes in kidney gene expression following TWEAK administration. At this point, some mRNA expression changes have already returned to baseline, as it is the case for MCP1 [5]. However, we now show that the expression of CD74 and its ligands MIF and DDT was persistently increased suggesting that TWEAK is one of the drivers of their increased expression in kidney disease. MIF expression is increased in progressive renal injury due to glomerulonephritis and renal transplant rejection [21,33–35]. MIF promoted podocyte injury and MIF targeting was protective in experimental glomerular disease [19,33]. This is most likely mediated by CD74 activation since CD74 targeting was also protective [19]. DDT, a member of the MIF cytokine superfamily, was recently described as a more selective CD74 agonist than MIF because it lacks the pseudo(E)LR motif present in MIF that mediates interaction with CXCR2 and CXCR4 [36] DDT was previously considered an enzyme that catalyzes the tautomerization and decarboxylation of D-dopachrome to 5,6-dihydroxyindole [37,38]. DDT is constitutively expressed in several mammalian tissues and is stored in macrophages [25,36,39]. As MIF, DDT may also be involved in regulating pro-inflammatory signaling events. DDT levels are elevated in septic, burn and cancer patients correlating with disease severity and clinical outcome [36,40]. However, DDT expression by kidney cells had not been previously described. For the first time, we have observed increased DDT expression by tubular cells in response to the systemic administration of a proinflammatory cytokine. In experimental endotoxemia, serum DDT levels are increased with a similar than MIF, and both proteins were detectable at similar concentration [36,41]. Thus, the DDT concentration used in the present report is clinically relevant, since circulating serum DDT has been described to range from 6 ng/ml in healthy individuals to 30 ng/ml under inflammatory conditions [36]. DDT blockade protected from lethal endotoxemia [16,36] and DDT binding to CD74/CD44 activates downstream pro-inflammatory intracellular pathways such as ERK1/2 MAPK signaling, AMP-activated protein kinase (AMPK), NFκB and β-catenin pathways in B cells, T cells and macrophages [16,42,43]. These pro-inflammatory actions are consistent with those observed in tubular cells in the present report. However, DDT binding to CD74 in liver and heart protects from ischemia-reperfusion and metabolic liver injury through activation of the AMPK pathway [44,45]. In this regard, DDT was recently reported to improve recovery of injured epithelial cells following kidney ischemia reperfusion [46].

There is limited information on the regulation of CD74 expression in renal cells. Abnormally high concentrations of certain metabolites (e.g. glucose and lyso-Gb3), TNF and interferon-γ increased CD74 expression in renal cells [21,47,48]. The present findings add TWEAK to the set of inflammatory cytokines that upregulate CD74 expression in kidney cells. The main site of CD74 expression is the perinuclear area, as observed in AKI immunohistochemistry. However, CD74 is also present at the cell surface as clearly demonstrated by flow cytometry in cultured tubular cells in presence of TWEAK and as also suggested by immunohistochemistry. Regulation of cell surface CD74 expression may have therapeutic consequences for the clinical use of agents currently in clinical development. The anti-CD74 antibody hLL1 milatuzumab, has received orphan drug status for the hematologic malignancies multiple myeloma and chronic lymphocytic lymphoma [49,50]. Milatuzumab binds to CD74, facilitating internalization of the antibody-CD74 complex, this is used to deliver conjugated antitumoral agents inside tumor cells with high CD74 expression, but not to normal cells with low CD74 levels [51]. Milatuzumab behaves as a CD74 antagonist, but activates antibody-dependent cellular cytotoxicity (ADCC) or complement-mediated cytotoxicity (CMC). Additionally, internalized toxins may kill tumor cells. However, nephrotoxicity is a potential complication of antitumoral anti-CD74 therapy, if kidney cells express high CD74 levels as observed in patients with kidney disease. As a clinical implication of the present findings, a state of systemic activation of the TWEAK/Fn14 system, exemplified by the systemic injection of TWEAK, may be associated to increased kidney CD74 expression, potentially sensitizing to cell death induced by milatuzumab and to nephrotoxicity. This may be of special concern for one of the indications under study, multiple myeloma, which frequently causes kidney disease. Unraveling the factors determining increased kidney cell expression of CD74 may help to design strategies to protect parenchymal cells from the toxicity of CD74 targeting anti-tumor therapies. Additionally, by localizing the expression of both ligands and the receptor, it may help unravelling the interactions between these molecules at the kidney level. Immunohistochemistry suggests potential secretion of MIF and DDT into differential compartments. MIF is localized mainly in the basolateral tubular cell pole, suggesting that it may be secreted to the interstitium, were it may interact with interstitial fibroblasts, leukocytes and tubular cells, while DDT is mainly found in the luminal pole, suggesting that it may contribute to signaling between different nephron segments, since as MIF, it was mainly found in proximal tubules, while distal tubules expressed high amounts of CD74.

The present findings may have clinical implications beyond kidney disease. Thus, CD74 plays a role in diverse diseases, either as a promoter of disease (e.g. glomerulonephritis) [19], as a protective molecule (e.g. liver fibrosis) [52] or as a therapeutic target that allows selective destruction of a tumor cell population (e.g. myeloma cells) [50]. In addition, CD74, interacts with amyloid precursor protein (APP), reducing the levels of beta amyloid peptides, probably by interacting with and derailing normal trafficking of APP [53]. Indeed, CD74 gene transfer reduced β-amyloidosis and improved cognitive function in a mouse model of Alzheimer's disease [54]. A better understanding of the drivers of CD74 expression may help to design therapeutic strategies aimed at modulating CD74 levels in the above mentioned conditions.

In conclusion, TWEAK upregulates CD74 and its ligands MIF and DDT in renal tubular cells in culture and in vivo. This may have functional consequences for kidney injury since DDT, at concentrations found in the circulation of inflamed patients, amplified the inflammatory response to TWEAK. In this regard, the present findings support the concept that DDT is a new player in tubular cell inflammatory responses.
